# The Rationale for Tissue Biopsy in the Era of Precision Hepatocellular Carcinoma Management

**DOI:** 10.3390/cancers18193115

**Published:** 2026-09-25

**Authors:** Ioanna-Maria Grypari, Dina G. Tiniakos, Maria Kalafateli, Eleni Theocharidou

**Affiliations:** 1 Pathology Department, Faculty of Medicine, School of Health Sciences, University of Ioannina, 45500 Ioannina, Greece; 2 Department of Pathology, Aretaeion Hospital, School of Medicine, National and Kapodistrian University of Athens, 11528 Athens, Greece; 3 Translational and Clinical Research Institute, Faculty of Medical Sciences, Newcastle University, Newcastle upon Tyne NE1 7RU, UK; 4 4th Department of Internal Medicine, Hippokration General Hospital, Aristotle University of Thessaloniki, 54124 Thessaloniki, Greece; 5 Division of Digestive Diseases, Department of Metabolism, Digestion and Reproduction, Faculty of Medicine, Imperial College London, London W2 1NY, UK; 6 Second Department of Internal Medicine, Hippokration General Hospital, Aristotle University of Thessaloniki, 54124 Thessaloniki, Greece

**Keywords:** hepatocellular carcinoma, tissue biopsy, surveillance, LI-RADS, histologic subtypes, molecular subtypes, prognosis, prediction

## Abstract

Hepatocellular carcinoma (HCC) is the most common liver cancer and usually develops on a background of cirrhotic liver. Early and accurate diagnosis enables potentially curative interventions. HCC surveillance, usually offered to patients at risk for HCC, such as those with cirrhosis, chronic hepatitis B infection, and previous diagnosis of HCC, is associated with earlier detection and improved survival. In these patients, the diagnosis can be established non-invasively without histological confirmation when the imaging-based LI-RADS criteria are met. Tissue biopsy is mandatory for establishing diagnosis in patients without cirrhosis or other risk factors for HCC, differentiating HCC from other malignant and non-malignant entities, especially when imaging features are not conclusive. In this review, we discuss the evolving role of tissue biopsy beyond establishing the diagnosis, in providing important prognostic information and enabling precise histopathological classification. Features such as poor differentiation, microvascular invasion, and a pattern of vessels encapsulating tumor clusters are linked to worse prognosis. HCC is characterized by heterogeneity, with different histological subtypes associated with distinct clinical and radiological features and distinct biology. Emerging data from molecular profiling and immune-microenvironment studies enable further classification into proliferative versus non-proliferative molecular subgroups and inflamed versus non-inflamed immune subtypes. This more refined characterization of HCC carries important implications not only for prognostication but also for predicting response to immunotherapy. In the future, the combination of imaging features that correlate with molecular signatures, novel serum biomarkers, and liquid biopsy may provide less invasive tools for early detection, risk stratification, and guide personalized therapeutic strategies.

## Simple Summary

Liver cancer is one of the leading causes of cancer-related deaths worldwide, and hepatocellular carcinoma is its most common form. Unlike most cancers, it can often be diagnosed without taking a tissue sample in people with chronic liver disease. However, tissue biopsy remains essential in many patients and is becoming increasingly important because it can reveal features that help predict how cancer will behave and which treatments are most likely to work. In this review, we examine the current approach to diagnosing hepatocellular carcinoma and the changing role of tissue biopsy. We also discuss histological subtypes, as well as emerging technologies that may improve diagnosis and support more personalized treatment in the future, highlighting how combining different modalities with tissue-based analysis may lead to better patient care.

## 1. Introduction

According to the 2022 Global Cancer Observatory (GLOBOCAN) estimates, liver cancer is the sixth most commonly diagnosed cancer worldwide, with 865,269 new cases, and the third leading cause of cancer-related mortality, accounting for 757,948 deaths in 2022 [1]. The incidence and mortality rates are approximately two- to three-fold higher in men than in women across most regions of the world [1]. Hepatocellular carcinoma (HCC) represents the predominant histological type, accounting for more than 75% of all primary liver cancers [2]. The global etiological landscape of HCC is also evolving, with a shift from predominantly viral hepatitis-related disease toward liver injury associated with metabolic dysfunction and alcohol misuse [2].

Early and accurate diagnosis of HCC is pivotal to patient management, as it enables the identification of tumors at a stage when potentially curative therapies, including surgical resection and liver transplantation, are feasible [3]. These interventions achieve 5-year survival rates approaching 70%, whereas advanced-stage HCC remains associated with a median survival of less than 1 year despite significant therapeutic advances [4,5]. Once the diagnosis is established, accurate staging is essential for prognostication and treatment allocation. Unlike other solid malignancies, prognosis in HCC is determined not only by tumor burden but also by the severity of the underlying liver disease and the patient’s performance status [3]. Consequently, contemporary staging systems integrate tumor stage, liver function, and functional status, with the Barcelona Clinic Liver Cancer (BCLC) classification being the system recommended by the European Association for the Study of the Liver (EASL), as it directly links disease stage to evidence-based treatment recommendations [6,7,8,9,10].

Unlike most solid malignancies, HCC can be diagnosed non-invasively in selected at-risk populations based on characteristic imaging findings, without the need for histological confirmation [3]. This approach is applicable only to individuals with a high pre-test probability of HCC [3,11,12]. Conversely, patients without high-risk factors and those with indeterminate imaging findings generally require histological confirmation to reliably distinguish HCC from benign lesions or other primary and secondary liver malignancies [3]. Moreover, the increasing recognition of the biological heterogeneity of HCC has expanded the role of tissue biopsy beyond diagnosis, as histopathological and molecular characterization can provide clinically relevant information for prognostic assessment, therapeutic decision-making, and the development of precision oncology strategies.

Although non-invasive imaging has revolutionized the diagnosis of HCC, tissue biopsy continues to play a pivotal and expanding role in clinical practice. This review examines the contemporary diagnostic framework of HCC, highlighting the indications and limitations of biopsy and its contribution to histopathological and molecular characterization, prognostic assessment, and personalized therapeutic decision-making. Finally, it discusses emerging technologies that are expected to further reshape the diagnostic landscape of HCC.

## 2. At-Risk Populations and HCC Surveillance

HCC develops predominantly in the setting of chronic liver disease, with approximately 80–90% of cases arising in cirrhotic livers irrespective of the underlying etiology. Nevertheless, a substantial proportion of HCCs occur in the absence of cirrhosis, particularly in patients with chronic hepatitis B (HBV) infection and, increasingly, in those with metabolic dysfunction-associated steatotic liver disease (MASLD) [1]. As the global burden of MASLD continues to increase [2], the absolute number of non-cirrhotic HCCs is expected to rise, posing new challenges for early detection and definitive diagnosis.

Because the risk of HCC varies considerably according to the underlying chronic liver disease and fibrosis stage, surveillance is recommended only for populations in whom the expected annual incidence justifies the costs and potential harms of screening [3]. The primary aim is to detect tumors at an early stage when curative therapies remain feasible. Current surveillance recommendations for the major at-risk populations are summarized in Table 1. In brief, surveillance is recommended for all patients with cirrhosis who are candidates for HCC-directed treatment, irrespective of disease etiology, and for selected non-cirrhotic patients with chronic HBV infection who are at intermediate or high risk of HCC, as identified by clinical risk factors and validated prediction models such as the PAGE-B score [3].

By contrast, routine surveillance is not currently recommended for most non-cirrhotic patients with MASLD or for individuals with chronic hepatitis C (CHC) who have achieved sustained virological response but retain advanced fibrosis, as existing risk-stratification models have not yet been sufficiently validated for routine clinical practice [13,14].

The clinical benefit of surveillance in appropriately selected populations is well established. In a recent meta-analysis of 145,396 patients with cirrhosis (59 studies), HCC surveillance was associated with improved early-stage detection (RR 1.86, 95% CI 1.73–1.98), increased use of curative treatment (RR 1.83, 95% CI 1.69–1.97), and prolonged overall survival (HR 0.67, 95% CI 0.61–0.72) after adjusting for lead-time bias. While the rate of surveillance-associated physical harm from false-positive or indeterminate results ranged from 8.8% to 27.5% across four studies, these events were mostly mild in severity [4].

Abdominal ultrasound performed every six months remains the cornerstone of HCC surveillance because of its wide availability, favorable safety profile, and cost-effectiveness [3,15]. While the addition of serum alpha-fetoprotein (AFP) modestly improves the sensitivity for early-stage HCC detection (from 52% to 74%), it is accompanied by a slight reduction in specificity [3], causing its routine use to vary among international societies [3,15]. Despite widespread clinical endorsement, the performance of ultrasound is operator-dependent and suboptimal in a significant proportion of patients, particularly those with obesity, ascites, steatotic liver disease, or markedly nodular cirrhotic livers, highlighting an important unmet clinical need [3]. Given these limitations, alternative surveillance strategies, including abbreviated magnetic resonance imaging (aMRI) [16,17], novel circulating biomarkers, and risk-based surveillance algorithms, are currently under active investigation [3,15].

Importantly, the identification of at-risk populations not only determines eligibility for surveillance but also establishes the pre-test probability of HCC, which directly influences the diagnostic algorithm. While the high prevalence in patients with cirrhosis or chronic HBV enables reliable imaging-based diagnosis, this framework is not applicable to lower-risk populations, in whom the reduced pre-test probability substantially increases the likelihood of false-positive imaging findings. Consequently, tissue confirmation is frequently required, making accurate risk stratification the critical first step in the contemporary diagnostic algorithm.

## 3. Non-Invasive HCC Diagnosis

Accurate diagnosis and staging are pivotal for guiding effective therapeutic decisions in HCC management. The differential diagnosis includes both benign (regenerative nodule, adenoma, focal nodular hyperplasia) and malignant liver tumors (cholangiocarcinoma, combined hepatocellular-cholangiocarcinoma (combined HCC-CCA), liver metastases). The diagnosis of HCC can be established on non-invasive imaging-based criteria alone, without histological confirmation, in appropriately selected at-risk patients, specifically those with cirrhosis, chronic HBV, and a previous diagnosis of HCC, while in adult patients without cirrhosis and in pediatric patients, a biopsy is mandatory [3,15]. The diagnosis should be confirmed by lesional biopsy in high-risk patients when the imaging-based criteria are not met [3].

Non-invasive diagnosis is feasible due to the unique vascular changes that occur in the process of HCC carcinogenesis, and that can be captured on dynamic contrast-enhanced imaging. HCC carcinogenesis follows a stepwise model, in which progressive accumulation of genetic and epigenetic alterations transforms normal hepatocytes into malignant cells, usually within a background of cirrhosis [3]. The transition from a benign regenerative nodule to advanced carcinoma follows the sequential stages of regenerative to dysplastic nodule (low-grade to high-grade), early, and then advanced HCC. In this transition from benign to dysplastic to malignant, major changes in vascularization occur. Normal liver tissue and regenerative nodules receive blood primarily from the portal vein. In contrast, in advanced HCC, the primary blood supply shifts to the hepatic artery via arterial neovascularization. Vascular changes are the hallmark used to identify HCC on dynamic contrast-enhanced imaging [18].

The liver imaging reporting and data system (LI-RADS) is a standardized system for classifying liver lesions in patients at high risk for HCC, using magnetic resonance imaging (MRI), computed tomography (CT), or contrast-enhanced ultrasound (CEUS) [11]. HCC can be diagnosed with high accuracy when the LI-RADS criteria are met in patients with cirrhosis, chronic HBV infection (and PAGE-B ≥ 10 [19]), and prior diagnosis of HCC. The LI-RADS criteria do not apply and should not be used in patients without cirrhosis and those with cirrhosis of vascular etiology (Budd–Chiari syndrome, chronic portal vein thrombosis, cardiac cirrhosis). Both the American Association for the Study of Liver Diseases (AASLD) and the recent EASL guidance support the use of LI-RADS to standardize non-invasive diagnosis in at-risk populations [3,15].

Two diagnostic algorithms have been developed, one for CT/MRI and another for CEUS (latest version released in 2018) [11]. LI-RADS diagnostic algorithm for CT/MRI relies on five major imaging features (Table 2) to categorize liver lesions on a scale from LR-1 (benign) to LR-5 (definite HCC). Arterial-phase hyperenhancement and washout on the portal venous and/or delayed phase are the characteristics most strongly associated with HCC. LI-RADS criteria accuracy decreases in lesions < 2 cm; therefore, different criteria apply to lesions 10–19 mm (Table 2). LI-RADS has introduced two further categories, the LR-TIV (“tumor in vein”) for macrovascular venous invasion, and the LR-M category for probably or definitely malignant lesions, but not necessarily HCC. The CEUS diagnostic algorithm assigns liver lesions to the same categories, but uses different major imaging features (Table 2). It applies to CEUS examinations performed with pure blood-pool agents, such as SonoVue^®^ (SonoVue Bracco S.p.A. (Milan, Italy)), but not with combined blood-pool and Kupffer-cell agents, such as Sonazoid^®^ (Sonazoid GE Healthcare AS (Oslo, Norway)).

While all three imaging modalities show high specificity for diagnosing HCC, CEUS is associated with significantly lower sensitivity and cannot be recommended as a first-line test. In a prospective multicentre study, 544 nodules 10–30 mm in size were assessed in 381 patients using CEUS, CT and MRI performed within a month [20]. For 10–20 mm nodules, the sensitivity and specificity for the diagnosis of HCC were 71% and 83% for MRI, 68% and 77% for CT, and 40% and 93% for CEUS, respectively. For 20–30 mm nodules, the sensitivity and specificity were 72% and 89% for MRI, 72% and 94% for CT, and 53% and 92% for CEUS, respectively. Although a meta-analysis suggested higher sensitivity for MRI than CT (82% vs. 66%) with similar specificity (92% vs. 91%) for diagnosing HCC [21], both modalities are equally recommended by EASL and AASLD, considering that MRI is associated with higher cost and technical complexity. CEUS can be used as a second-line test when CT or MRI are inconclusive.

Hepatic steatosis has a negative impact on detection and characterization of liver lesions, particularly with CT [22]. MRI is more accurate and should be preferred in this context [23]. Liver-specific (hepatobiliary) MR contrast agents (HBA) have been a major development in MR imaging. HBA, contrary to extracellular contrast agents (ECA), are taken up by functioning hepatocytes, while their uptake decreases early in hepatocarcinogenesis, a property that accounts for the hypointensity of liver nodules on the hepatobiliary phase. HBA-MRI has higher sensitivity in detecting liver nodules < 2 cm, but the specificity for HCC diagnosis is significantly lower than ECA–MRI (68% vs. 83%) [24], which should be preferred over HBA-MRI. HBA-MRI has a potential role for local tumor staging in patients who are candidates for curative treatment, such as resection, transplantation, or ablation, as it can detect additional small lesions, resulting in more accurate BCLC staging [25].

The LI-RADS classification provides information on the risk of HCC and can guide the management strategy (Figure 1) [15,26]. A biopsy is not required for LR-5 lesions, and these patients should undergo staging with contrast-enhanced CT of the chest, abdomen, and pelvis. Measurement of AFP or other blood-based tests, such as des-gamma carboxy-prothrombin (DCP) and lectin-bound AFP (AFP-L3), is not part of the diagnostic strategy, but it can provide prognostic information once a definite diagnosis of HCC has been made. Elevated AFP is associated with biologically aggressive disease, including poorer differentiation and increased likelihood of vascular invasion, and higher AFP levels (≥400 ng/mL) are linked to worse outcomes after resection, locoregional therapy, systemic therapy, and liver transplantation [27], but normal levels do not preclude aggressive disease.

Overall, non-invasive HCC diagnosis is an evidence-based, highly useful approach that allows many at-risk patients to proceed to staging and treatment without biopsy. Its efficiency depends on three conditions: the patient must belong to a high-risk population (cirrhosis, chronic HBV infection, and previous HCC), the imaging must be technically adequate (CT, MRI, or CEUS), and the lesion must meet the LI-RADS criteria. When those conditions are not met, biopsy or repeat imaging is mandatory. This non-invasive strategy has limitations that need to be considered: it does not apply to patients without cirrhosis and those with vascular disorders of the liver; the accuracy of LI-RADS criteria is decreased in lesions < 2 cm (dysplastic nodules and small HCC can overlap substantially) and in patients with chronic HBV and PAGE-B score < 10; APHE is non-specific, with diverse lesions mimicking HCC; technical dependence (motion, mistimed contrast phases) can interfere with imaging interpretation; MRI is more technically complex, and CEUS is operator-dependent. Non-invasive imaging-based assessment alone is insufficient in cases of indeterminate or atypical lesions, in non-cirrhotic or low-risk HBV patients, in small lesions < 1–2 cm, when imaging cannot differentiate HCC from other hepatic malignancies, or when imaging modalities show discordant findings. In these cases, a lesion biopsy is often required for histological confirmation. Importantly, there are no imaging features that can predict outcomes. HCC is characterized by distinct molecular subgroups and pathological subtypes, each with unique prognostic implications, which can guide individualized therapeutic decision-making. Current research is focused on novel methods incorporating artificial intelligence and radiomics that provide functional and quantitative parameters in addition to morphological information, and aim to predict tumor grade, subtype, and microvascular invasion [28,29,30]. Currently, this important prognostic and potentially predictive information can only be provided by tumor biopsy.

## 4. Tissue Biopsy

Even though imaging recognizes suspicious focal liver lesions with significant accuracy, histopathological confirmation remains a cornerstone of precision medicine [3].

Biopsy becomes particularly valuable in the following scenarios:

- **Atypical imaging presentation**—confirms diagnosis and excludes other neoplastic lesions when radiological features are non-specific, which occurs in up to 40% of HCCs [31,32].
- **Complex differential diagnosis**—distinguishes HCC from benign, precancerous, or other malignant entities, particularly relevant given the varying differential diagnoses in cirrhotic versus non-cirrhotic liver. In cirrhotic liver, benign lesions such as large regenerative nodules [33] and focal nodular hyperplasia-like nodules must be excluded [34]; the differential diagnosis includes precancerous lesions, such as low- and high-grade dysplastic nodules, and non-HCC malignancies such as mass-forming intrahepatic cholangiocarcinoma (CCA), combined HCC-CCA, undifferentiated primary liver carcinoma, and metastatic carcinoma [34]. In non-cirrhotic liver, differential diagnosis includes focal nodular hyperplasia, hepatocellular adenoma, lobular or segmental hyperplasia, and focal fatty change, as well as other entities such as inflammatory pseudotumor, bile duct adenoma, angiomyolipoma, and pyogenic abscess [33,34]. Given the major therapeutic differences among these entities, correct diagnosis is essential for proper management.
- **Histopathological subtyping**—identifies distinct clinicopathological and molecular subtypes and intratumoral heterogeneity that imaging may miss [35,36]. Calderaro et al. proposed dividing HCC into well-defined morphomolecular groups, in which specific histologic appearances correlate with particular genetic alterations and transcriptomic signatures [37], enabling stratification by tumor biology, clinical outcome, and potential response to targeted therapies.
- **Prognostic stratification**—provides histological differentiation (grade), vascular invasion, and growth pattern linked to poorer prognosis; data that inform multidisciplinary, personalized treatment planning [32,38].
- **Advanced-stage disease (e.g., stage IV-B)**—offers a statistically significant benefit, likely because biopsy provides predictive data—through molecular studies—for immunotherapy or systemic therapy, according to a recent Korean study [39].
- **Transplant candidacy**—establishes diagnosis in patients meeting transplantation criteria [32].
- **Clinical trial eligibility**—selects candidates for trials and systemic/transplant therapies, while generating molecular and immune-microenvironment data that supports biomarker development [32,36,40].

The main limitations of tissue biopsy are associated with the invasiveness of the procedure, the risk of complications, and the sampling bias. Although serious complications are uncommon (pain, bleeding, perforation of adjacent organ), they can rarely be life-threatening. Needle track neoplastic cell seeding is a significant risk, with an estimated incidence of 1.62–2.7% in older meta-analyses but with no recurrence after local excision. However, more recent studies showed that with the use of coaxial cutting or smaller diameter needles and expert operators, seeding rate may drop to 0–0.5% [32,41,42]. Importantly, the small sample size obtained with a needle biopsy may not be representative of the tumor heterogeneity [38]. Furthermore, biopsy may be contraindicated or technically challenging in cirrhotic patients with coagulopathy, thrombocytopenia, or ascites, sometimes requiring alternative approaches such as transjugular biopsy [43,44,45]. A false-negative or non-diagnostic result does not exclude HCC and may necessitate repeat biopsy or reliance on imaging follow-up, potentially delaying diagnosis and treatment [32].

## 5. Histological Subtypes of HCC

Approximately 35% of HCCs can be classified into a distinct morphological subtype; the remainder are designated HCC, not otherwise specified (NOS). The most common histological growth patterns that are encountered in HCC NOS are solid, trabecular, and pseudoglandular (acinar) [35].

Histological HCC subtypes according to the 6th edition of the WHO classification of Digestive System Tumors [35] are described in Table 3 and further discussed below. Representative images from selected HCC subtypes are demonstrated in Figure 2.

### 5.1. Fibrolamellar HCC

Fibrolamellar HCC has distinctive morphology and clinical behavior compared to classical HCC. It accounts for ~1% of primary liver carcinomas [48], typically arising in young adults without cirrhosis or liver disease, with equal sex distribution [48,49]. It disseminates through lymph nodes [49], and patients often develop distant metastases [48]. Serum AFP levels remain normal, so they cannot serve as a biomarker for this tumor.

There is no pathognomonic imaging pattern, though it typically presents as a large hepatic lesion with a central scar and heterogeneous enhancement [31]. On MRI, it is usually hypointense on T1 and hyperintense on T2, while the central scar is hypointense on both sequences [50,51]. On gadoxetic acid–enhanced MRI, inhomogeneous arterial-phase enhancement with hepatobiliary-phase hypointensity is observed [50], and these tumors typically lack a distinct capsule, implying an infiltrative growth pattern [50]. It can be mistaken for other scar-bearing focal liver lesions—focal nodular hyperplasia, hepatocellular adenoma, hemangioma—as it affects young people without chronic liver disease [31], which explains the significance of biopsy for guiding management.

Main histological features of fibrolamellar HCC are described in Table 3 [48,52]. Nuclear pleomorphism is mild (Figure 2B) and mitoses are sparse [48]; cytoplasmic inclusions, including fibrinogen-containing pale bodies, are common, and fat deposition or bile production may be seen [48]. Immunohistochemically, there is unusual co-expression of classical hepatocytic markers (HepPar1) with epithelial membrane antigen, keratin 7 (K7), and CD68 [52,53].

Diagnosis is confirmed by detecting the pathognomonic *DNAJb1-PRKACA* fusion via FISH or RNA-based NGS [52]. mTORC1/2 pathways are also implicated, particularly mTORC1 activation linked to FGFR1 [53], though mTORC1 inhibition has shown no clinical benefit [54]. Conversely, a phase I trial combining ipilimumab and nivolumab with a peptide vaccine targeting the *DNAJB1-PRKACA* fusion achieved disease control in 75% of patients and objective radiological response in 25% [49,55]— a promising step toward personalized therapy for this tumor.

Standard treatment is surgical resection; no systemic therapy is specifically approved, though cytotoxic chemotherapy trials are ongoing [49]. Complete resection with lymphadenectomy yields 5-year overall survival up to 70%; liver transplantation is an alternative when resection is not feasible [48]. Accurate pre-operative diagnosis is therefore crucial for treatment selection. Emerging data suggest serum procalcitonin, elevated in these patients and correlating with disease progression, as a potential biomarker for diagnosis and follow-up, since it is not overexpressed in other primary liver malignancies [49].

### 5.2. Macrotrabecular-Massive HCC

Macrotrabecular-massive HCC (MTM–HCC) (Figure 2C) [36] has a reported incidence of 8.5–12% [47].

On MRI, MTM–HCC shows internal/diffuse arterial-phase hypoenhancement (reflecting common intratumoral necrosis) with peripheral hyperenhancement or spot-like hyperenhancing foci [56]. On gadoxetic acid–enhanced MRI, MTM–HCC is suggested by ≥20% hypovascular tumor component in the arterial phase (95–97% negative predictive value), or ≥50% hypovascular tumor combined with more than two ancillary findings (intratumoral arteries, arterial-phase peritumoral enhancement, non-smooth margin)—the latter highly specific (94–96%) and linked to poor outcome [57].

Calderaro et al. analyzed 343 HCCs and found that MTM–HCCs frequently harbor TP53/ATM mutations and FGF19 amplification, with upregulated angiogenesis genes; this pattern was more commonly associated with HBV infection and higher AFP. In addition, MTM–HCC demonstrated increased microvascular/macrovascular invasion and satellite nodules [37]. Subsequent studies confirmed shorter overall and recurrence-free survival [58,59] and identified the MTM–HCC subtype as an independent predictor of recurrence [58], though satellite nodules were not significantly associated with MTM–HCC in one study [59]. Ziol et al. further showed that the macrotrabecular pattern predicts higher recurrence after radiofrequency ablation versus resection [58]. Given its prognostic weight, any degree of macrotrabecular architecture should be reported on both biopsy and resection.

### 5.3. Steatohepatitic HCC

Steatohepatitic HCC is the most common subtype, comprising 10–20% of cases [59,60], predisposed by metabolic syndrome and alcohol misuse [60,61,62]. It coexists with type 2 diabetes and a steatotic/steatohepatitic background liver [63]. Histology shows steatosis, tumor cell ballooning, Mallory-Denk bodies, pericellular “chicken-wire” fibrosis, and intratumoral inflammation (Figure 2D) [51,55]. Ballooning is the most specific feature for steatohepatitic HCC, followed by pericellular fibrosis [64].

Calderaro et al. assigned this subtype to morphomolecular Group 4 (low mitotic activity, chromosomal stability) [37], molecularly marked by IL-6/JAK/STAT activation rather than the WNT/β-catenin, TP53, or TERT alterations typical of conventional HCC; CRP expression serves as a surrogate marker of IL-6 pathway activity [37]. Prognosis is similar [62] to conventional HCC [65], with less microvascular invasion or satellite nodules in some series [37]. The percentage of the steatohepatitic component in an HCC does not impact patient survival.

Not all steatotic HCCs can be designated as the steatohepatitic subtype [62], and whether steatotic HCCs lacking the full histological picture share its prognosis remains unclear [66]. Differential diagnosis on tumor biopsy is challenging because steatohepatitic HCC shares features with steatohepatitis and the two may coexist; features favoring malignancy include portal tract invasion (may not be sampled in all biopsies), unpaired arterioles, absent portal tracts, and increased plate cellularity—reticulin loss should be interpreted cautiously, as it can also occur in severe steatosis and steatohepatitis [67]. Clinicoradiologic correlation helps, as a discrete, ill-defined mass favors HCC, while absence of a mass favors steatohepatitis [68].

Imaging often shows fat-related features—ultrasound hyperechogenicity, CT fat attenuation, MRI T1 hyperintensity, and dual-echo signal loss [50]. A study comparing steatohepatitic HCC with simple steatotic HCC found combined MRI/clinical fatty-change assessment highly specific (97%) but only moderately sensitive (53–60%), highlighting that histopathology remains the only definitive method of distinction [63]. Nevertheless, definitive differentiation between steatohepatitic HCC and HCC with simple steatosis remains possible only through histopathological examination [63]. As the incidence of MASLD rises globally, further studies clarifying the biology and management implications of steatotic HCC subtypes are needed.

### 5.4. Scirrhous HCC

Scirrhous HCC is the fifth most prevalent subtype (~4%) [59,69,70].

Immunohistochemically, keratin 7 is expressed in 40% of cases and keratin 19 (K19) in 16% of cases [60], while conventional hepatocellular marker expression (HepPar-1, arginase-1, and glypican-3) is retained, at frequencies comparable to conventional HCC [60,71]. Combined assessment of hepatocellular and biliary/progenitor markers helps distinguish scirrhous HCC from CCC, its main differential diagnosis given shared K19/EpCAM expression [71]. Molecularly, it differs from conventional HCC via TSC1/TSC2 mutations and TGF-β pathway activation [37,69].

The desmoplastic tumor stroma (Figure 2E) is responsible for imaging appearance that mimics intrahepatic CCC or desmoplastic metastases: peripheral rim enhancement (arterial/portal phases) with progressive central enhancement and no washout, targetoid DWI appearance, confluent multinodular capsule-free growth, and peripheral/subcapsular location with capsular retraction—though none are pathognomonic, as similar features occur in CCC, combined HCC-CCC, and fibrolamellar HCC [31,50,51]. Features favoring scirrhous HCC over CCC include a peritumoral capsule, intratumoral fibrous septa, and T2 central hypointensity [72], plus >20% arterial hyperenhancement on gadoxetic acid MRI with DWI hyperintensity [73].

Prognostic significance remains uncertain, though larger tumors may show more aggressive behavior (vascular invasion, infiltrative growth, shorter survival) [69]. A cohort of 30 scirrhous versus 120 non-scirrhous HCC patients showed similar overall survival but significantly higher 2-year recurrence in the scirrhous group after curative therapy [74].

### 5.5. Chromophobe HCC

Chromophobe HCC is uncommon (~3% of HCCs) [69] and was first recognized in the WHO 5th edition [60]. Data remain limited given its rarity, but it appears to occur predominantly in women without a specific predisposing condition [60,69,70]. The main diagnostic challenge is distinguishing primary chromophobe HCC from metastatic renal chromophobe carcinoma; the latter expresses K7 and CD117/CD10, all three absent in chromophobe HCC [70].

Molecularly, chromophobe HCC is associated with the alternative lengthening of telomeres (ALT) pathway—a telomerase-independent, homologous-recombination-based mechanism detectable by telomere FISH, occurring without TERT promoter mutations or rearrangements [60,70]. No significant prognostic difference from other HCC has been reported to date [60,62,69].

### 5.6. Clear-Cell HCC

Clear-cell HCC [69] accounts for 3–7% of HCCs. It is typically well-to-moderately differentiated with glycogen-rich neoplastic cells [62]; macrovesicular steatosis may also complicate diagnosis [22]. Its pathogenesis remains poorly understood [60,62,69].

Differential diagnosis includes lipid-rich conventional HCC and other clear-cell tumors, notably metastatic clear-cell renal carcinoma (ccRCC) and angiomyolipoma [70]. Immunohistochemistry is key: clear-cell HCC expresses hepatocellular markers, while ccRCC is PAX8/CAIX-positive and lacks them [70]—an important distinction given its impact on management. Radiologically, it resembles conventional HCC, with no specific imaging features [51].

Clear-cell HCC has generally been associated with more favorable outcomes [59,69], though a single-institution study of 589 HCCs found an association with more frequent recurrence/metastasis [59]. The same group found IDH1 mutations by pyrosequencing in 20 clear-cell HCCs, not seen in other subtypes [75].

### 5.7. Lymphocyte-Rich HCC

This rare subtype (<1% of HCCs, occasionally higher in selected series [53,66], previously termed lymphoepithelioma-like HCC, was renamed in the WHO 5th edition [60]. The dense lymphocytic infiltrate—predominantly CD8+ T cells, sometimes with B cells and germinal centers [60,70]—can exceed tumor cell numbers in many fields; hepatocellular markers remain unchanged on immunohistochemistry. Differential diagnosis includes metastatic lymphocyte-rich carcinomas from the stomach, nasopharynx, and other sites.

Unlike its counterparts in other organs, this HCC subtype has no established EBV association and is typically microsatellite stable [62]. Clinically, it carries a more favorable prognosis, attributed to antitumoral CD8+ infiltration, though its precise molecular profile remains unknown [60,69].

### 5.8. Neutrophil-Rich HCC

This rare subtype, characterized by dense intratumoral neutrophilic infiltrate, was first recognized in the WHO 5th edition. Patients often show leucocytosis, elevated CRP, and elevated IL-6, reflecting systemic inflammation [69]. Prognosis is poorer than conventional HCC, with shorter survival [60].

A characteristic feature is tumor production of G-CSF, likely driving both the neutrophilic infiltrate and leucocytosis [70]. G-CSF immunohistochemistry can support diagnosis but is not routinely available in most laboratories [70].

## 6. Vessels Encapsulating Tumor Clusters Histological Pattern

Vessels encapsulating tumor clusters (VETC) is a prognostically significant histological pattern involving an Epithelial to Mesenchymal Transition (EMT)-independent metastatic mechanism [76], characterized by a continuous endothelial lining around HCC cell clusters (Figure 2F), with contributions from basement membranes, hepatic stellate cells, and pericytes forming a capillary-like network [76,77]. This vascular pattern is thought to enable metastasis via endothelial-coated multicellular microemboli [76,77] and is typically identified by CD34 (or CD31/CD105) immunostaining.

VETC pattern is more frequently detected in larger tumors (>5 cm) and is associated with multifocality, satellite formation, and AFP > 400 ng/mL; histologically, these tumors are usually poorly differentiated (grade 3), often macrotrabecular, with higher rates of microvascular invasion [76,77,78]. Molecularly, HCC with VETC pattern often shows Wnt/β-catenin activation and aligns with Calderaro’s G5–G6 subgroup (CTNNB1 mutations, diffuse glutamine synthetase expression) [37]. However, VETC-positive tumors are only a minority of G5–G6 HCCs and may represent a poorer-prognosis subset requiring further study [77]. Their microenvironment is relatively depleted of CD8+/CD3+ T cells compared to MTM–HCC [76,77,79], but enriched for PD-L1-positive cells [77], supporting the rationale for combined anti-angiogenic/checkpoint inhibitor strategies [78].

VETC is strongly linked to early recurrence and reduced overall/disease-free survival [78,80], with extent—not just presence—affecting prognosis: focal VETC carries intermediate risk relative to extensive versus absent VETC [77,81]. A recent study, evaluating VETC pattern in pre-operative HCC biopsies, revealed that this vascular pattern of invasion serves as a predictive biomarker in patients with advanced HCC, receiving atezolizumab and bevacizumab or durvalumab and tremelimumab [82]. In liver-transplant explants, VETC burden (rather than mere presence) predicts post-transplant recurrence risk [81]. As imaging has only limited-to-moderate predictive ability, biopsy remains the primary tool for identifying VETC-positive HCC: gadoxetic acid–enhanced MRI arterial-phase irregular rim enhancement correlates with VETC and adverse features but with modest specificity (55%); dynamic CT offers moderate predictive performance (AUROC 0.69–0.72), improved by incorporating tumor size (>5 cm) and necrosis; contrast-enhanced ultrasound hypervascularity and increased PET/CT FDG uptake may also help, though clinical utility remains limited by tumor heterogeneity and interobserver variability [76].

## 7. Mutational Landscape in HCC

Hepatocarcinogenesis follows a stepwise molecular sequence: *TERT* promoter mutations occur early, followed by *CTNNB1* activation, with *TP53* mutations typically later [83]. *TERT* alterations can help distinguish hepatocellular adenoma from HCC but occur even in premalignant dysplastic nodules, limiting specificity; *CTNNB1*/*TP53* assessment improves this distinction, as these are rarely seen in dysplastic nodules [83,84].

The 2025 French Genomic Medicine Initiative’s genomic/transcriptomic analysis of 80 HCC patients found *TP53* mutations in 46%, *TERT* mutations in 44%, and *CTNNB1* alterations in 20% [84], consistent with previous literature [84,85,86].

Calderaro et al. combined morphology with molecular profiling to define proliferative and non-proliferative subgroups in 343 resected HCCs, based primarily on *TP53*/*CTNNB1* status [37]. The proliferative HCC subgroup (*TP53*-mutated, poorly differentiated, high AFP) divided into three subgroups: Group 1 (*RPS6KA3* mutations, clear-cell morphology, K19/EpCAM/phospho-ERK expression, female predominance); Group 2 (additional *ATM*/*AXIN1* alterations, sarcomatoid areas shared with G1); Group 3 (linked to hereditary hemochromatosis, *TSC1/TSC2* mutations, *FGF19* amplification, macrotrabecular/solid growth, macrovascular invasion, pleomorphism/multinucleation, phospho-S6 positivity) [37].

The non-proliferative HCC subgroup was predominantly well-differentiated and was subdivided chiefly by *CTNNB1* status. HCC in Groups 5/6 (*CTNNB1*-mutated) showed microtrabecular growth, cholestasis, minimal inflammation, nuclear β-catenin with diffuse glutamine synthetase staining, and frequent *ARID1A* loss. HCCs lacking both *CTNNB1* and *TP53* mutations formed the inflammatory Group 4, linked to the steatohepatitic subtype, typically small without satellite nodules or vascular invasion, with prominent inflammatory infiltrate and CRP expression.

Immune-microenvironment studies further divide HCC into inflamed and non-inflamed categories [87]; before this, only ~25% of cases could be classified as inflamed due to incomplete understanding of predictors of immunotherapy response [88]. Montironi et al. analyzed 240 HCC samples by multi-omics, developing a 20-gene signature to identify inflamed tumors, validated in cohorts totaling 660 patients [87]. They confirmed the established immune class (22%) and proposed a new Immune-like subclass (~15%) marked by enhanced interferon signaling, cytolytic activity, and diverse T-cell populations—together forming an expanded “Inflamed” class (~35% of cases), providing biological rationale for checkpoint inhibitor efficacy in this group [87].

The non-inflamed group split into two mutually exclusive subclasses: “Intermediate” (~43%, TP53-mutated with chromosomal losses) and “Excluded” (20%, CTNNB1-mutated, WNT/β-catenin activation, PTK2 overexpression) [87]. Notably, a subset of WNT-activated HCCs showed inflammation/immune activation (CCL5, CXCL10, enhanced MHC-I, denser infiltrate) with hypomethylated antigen-presenting genes, suggesting epigenetic involvement [87].

This classification highlights HCC’s heterogeneity and its potential as a clinical biomarker. β-catenin activation, for instance, can be found in both inflamed and non-inflamed HCCs: its presence sometimes correlates with diminished response to immunotherapy, while in other cases it identifies tumors with potential benefit.

## 8. Therapeutic Implications of Molecular Characterization of HCC

Molecular characterization identifies actionable alterations that guide personalized therapy. Campani et al., in a cohort of 120 primary liver malignancies (80 HCC), found that 59% harbored potentially targetable alterations; among 22 patients receiving molecularly guided therapy, 23% achieved disease control with prolonged progression-free survival [84].

Tumor immune microenvironment characterization also informs treatment response prediction. GO30140/IMbrave150 trial analyses showed atezolizumab-bevacizumab efficacy depends on tumor microenvironment features: greater benefit correlated with CD8+ infiltration, PD-L1 expression, and T-effector gene signatures, while reduced benefit correlated with oncofetal gene expression (AFP, GPC3) and regulatory T-cell (Treg) infiltration [89]. Bevacizumab benefit was greatest with high VEGFR2/KDR expression, myeloid inflammatory signatures, and Treg infiltration [89]—suggesting anti-VEGF and anti-PD-L1 therapy act synergistically on angiogenesis and immunosuppression.

The clinical significance of *CTNNB1* mutations remains inconclusive [90]. The KEYNOTE-240 trial showed no relationship between ctDNA, *CTNNB1* mutations (concordant with whole exome sequencing/WES), and outcomes with pembrolizumab or placebo [91], and no association between *CTNNB1* mutation and patient survival [92]. However, low CTNNB1 mRNA expression correlated with improved outcomes after first-line checkpoint inhibitor/tyrosine kinase inhibitor therapy, and expression levels correlated with DNA repair, immunity, neuronal signaling, and angiogenesis pathways [92]—warranting prospective validation as a predictive biomarker.

Overall, a combination of genomic and immunologic tumor profiling may identify future biomarkers predictive of therapeutic response, advancing personalized treatment and precision medicine in HCC.

## 9. Radiology-Pathology Correlation

Certain imaging phenotypes—hepatobiliary-phase hyperintensity on gadoxetic acid MRI in CTNNB1-mutated HCC, or targetoid features in specific subtypes—may suggest molecular or pathological characteristics, but none are sensitive or specific enough to replace histopathology.

CTNNB1-mutated HCC shows hepatobiliary-phase hyperintensity (strongly linked to OATP1B3 overexpression, with good diagnostic performance), higher ADC values, and lower DWI contrast-to-noise ratio [50].

HCCs expressing progenitor markers show a non-expanding growth pattern, progressive/persistent enhancement, lower likelihood of nodule-in-nodule appearance, higher DWI signal, and lower hepatobiliary-phase signal [93], which is insufficiently specific to replace biopsy.

Recent studies found no significant correlation between HCC histopathology (grade, microvascular invasion) and specific radiologic features [55]. However, combining multiple MRI-derived radiomic features may improve pre-operative prediction of microvascular invasion, and combining MRI texture with other imaging characteristics may predict immunological gene-expression patterns [94].

Radiomics—high-dimensional quantitative features from CT, MRI, and PET—is a promising future tool, already used to differentiate HCC from other malignant (CCC, combined HCC-CCA) and benign liver tumors (FNH, hemangioma, hepatocellular adenoma) with AUCs of 0.71–0.96, depending on lesion/modality [95]. It has also predicted pre-surgical histological grade (MRI- and CT-based AUCs up to 0.94, improved by combining with clinical features). Mixed radiomics/clinical models could predict microvascular invasion with AUCs up to 0.91 and further improved by peritumoral radiomic features [95]. However, most studies are retrospective, single-center, and rely on manual segmentation with limited external validation, so further standardization is needed before clinical adoption [95].

## 10. Perspectives

### 10.1. Artificial Intelligence

Zeng et al. developed an artificial intelligence (AI) model predicting the atezolizumab-bevacizumab response signature (ABRS) using 336 TCGA–LIHC HCC samples, validated in 225 resected and 157 biopsy HCC specimens, then further tested in 122 biopsy samples from patients treated with atezolizumab-bevacizumab [96]. Using spatial transcriptomics to compare AI-generated heatmaps with gene expression, they found ABRS expression correlated strongly with prediction scores (ABRS-p) (R = 0.62, *p* < 0.001) across all cohorts. Patients with ABRS-p-high tumors receiving atezolizumab-bevacizumab had significantly longer progression-free survival than ABRS-p-low patients. Spatial transcriptomics confirmed higher expression of ABRS-related and immune-infiltration genes in high-scoring tumors [96].

### 10.2. Role of Liquid Biopsy in HCC

Despite the central role of imaging, tissue biopsy, and serum biomarkers in HCC management, minimally invasive tools that improve early detection, prognostic stratification, and real-time monitoring remain needed. Liquid biopsy—based on circulating tumor cells (CTCs), cell-free DNA (cfDNA), and extracellular vesicles (EVs) [97]—offers this potential by capturing tumor-derived material released into the bloodstream.

Its principal current limitation is the low abundance of CTCs/cfDNA in early-stage disease, restricting diagnostic sensitivity. Nonetheless, both retain strong value in surveillance—monitoring treatment response, detecting minimal residual disease/recurrence, and identifying emerging resistance [38,97]. Finally, a key challenge that remains for EV-based liquid biopsy is improving the tissue specificity of HCC-derived EVs. Enhancing the ability to distinguish tumor-derived EVs from those released by normal tissues is essential for increasing the diagnostic accuracy and clinical utility of this approach [97]. The key challenge is improving tumor specificity to distinguish HCC-derived EVs from normal-tissue EVs. Standardizing CTC/EV isolation and detection protocols, reducing background noise, improving analytical sensitivity, and developing more cost-effective next-generation sequencing (NGS) technologies are required before including liquid biopsy in routine HCC management [97].

## 11. Conclusions

The diagnostic and management landscape of HCC relies on a precise balance between non-invasive imaging and tissue histopathology. For patients in well-defined high-risk groups, standardized imaging frameworks like the LI-RADS criteria allow for highly accurate, non-invasive diagnosis, streamlining the path to staging and treatment allocation. However, this non-invasive approach has clear boundaries; its diagnostic accuracy drops significantly in non-cirrhotic populations, atypical presentations, and lesions smaller than 2 cm.

As the global etiology of HCC shifts away from viral hepatitis toward MASLD, clinicians increasingly encounter non-cirrhotic and radiologically atypical tumors. In this evolving landscape, tissue biopsy has re-emerged as an essential tool for precision oncology. Beyond clarifying ambiguous imaging results and ruling out other malignancies, histopathological and molecular evaluation identifies distinct HCC subtypes. Recognizing these specific morphological and morphomolecular subgroups may provide critical prognostic insights and bridge the gap that currently exists in the route to individualized, biomarker-driven therapies in HCC. Future advancements combining AI, radiomics, and molecular profiling will likely further integrate imaging and pathology, ultimately refining early detection and individualized care for patients with HCC.

## Figures and Tables

**Figure 1:**
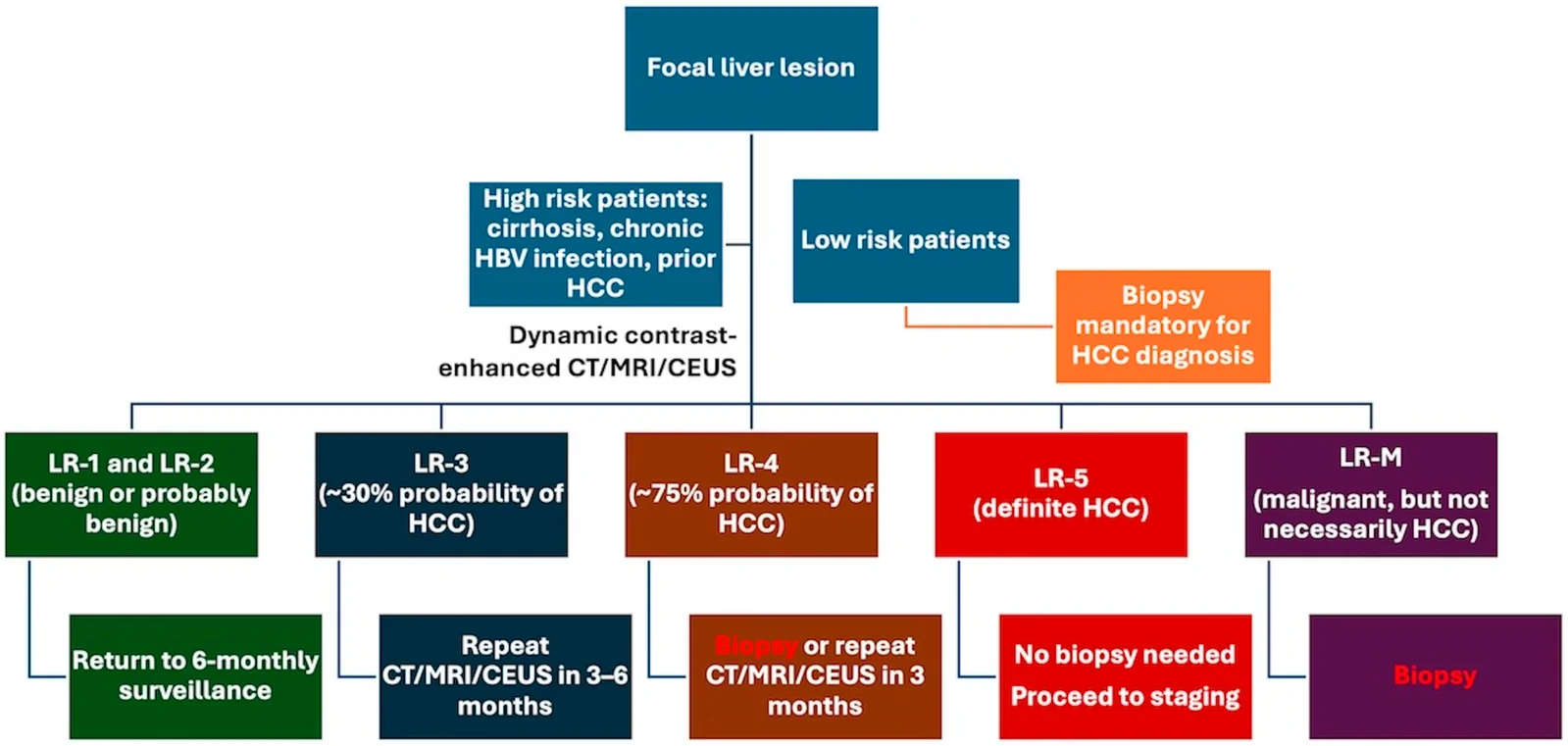
Probability of hepatocellular carcinoma and management strategy according to LI-RADS classification (modified from [15]). HBV, hepatitis B virus; HCC, hepatocellular carcinoma; CT, computed tomography; MRI, magnetic resonance imaging; CEUS, contrast-enhanced ultrasound.

**Figure 2:**
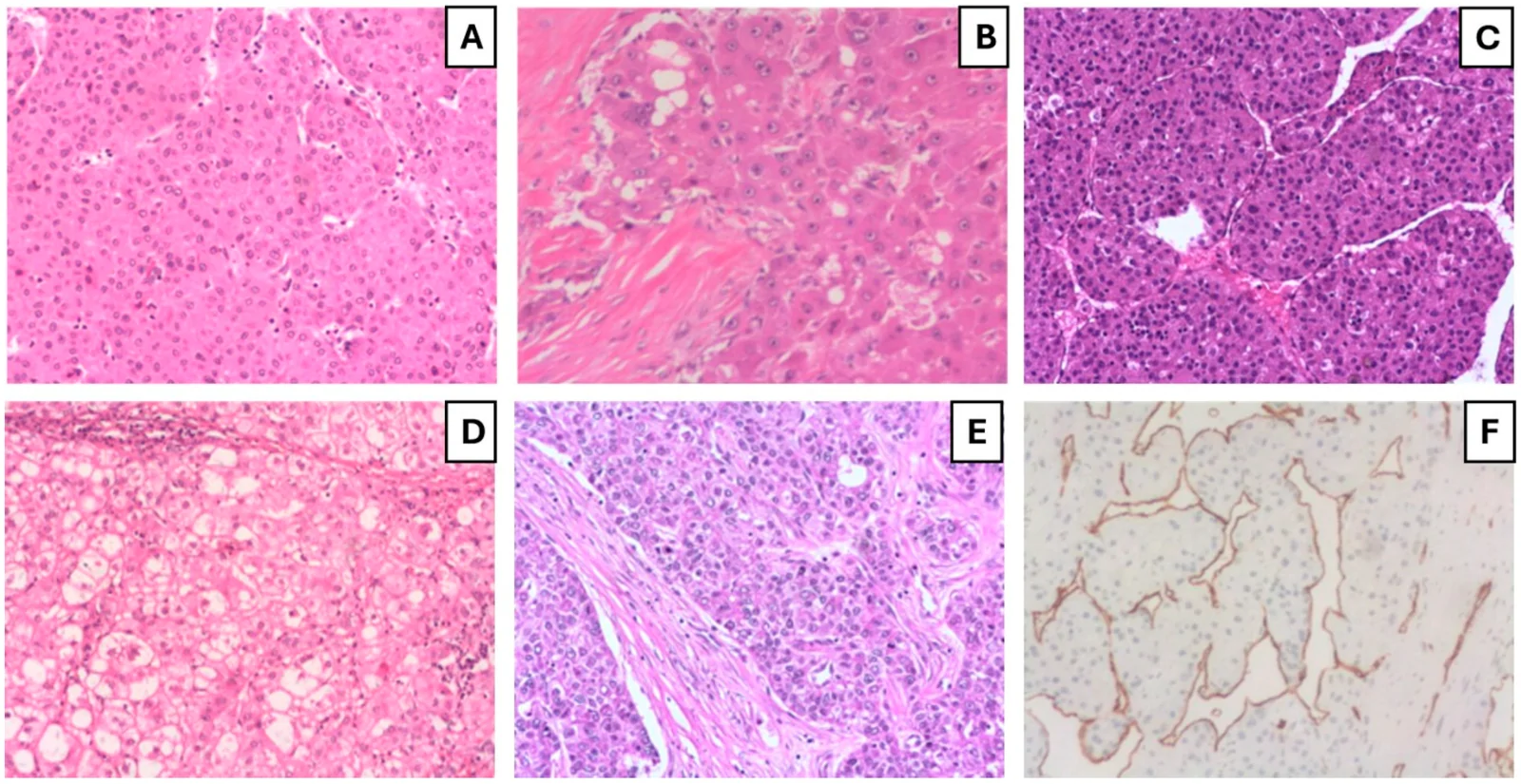
Representative images from selected types of hepatocellular carcinoma (HCC). (**A**). HCC, no special type (NOS), hematoxylin-eosin (H-E) x100; (**B**). Fibrolamellar HCC, H-E ×200; (**C**). Macrotrabecular massive HCC, H-E ×100; (**D**). Steatohepatitic HCC, H-E, ×200; (**E**). Scirrhous HCC, H-E ×100; (**F**). HCC with vessels entrapping tumor clusters (VETC) pattern, immunohistochemical stain CD34 ×100.

**Table 1:** Current recommendations for HCC surveillance according to patient population.

Population	HCC Risk	Current Surveillance Recommendation	Key Considerations
**Cirrhosis (any etiology)**	High	Recommended	Six-monthly ultrasound (±AFP according to guideline); for all patients eligible for HCC-directed treatment.
**Chronic HBV infection without cirrhosis**	Intermediate–high (selected patients)	Recommended in selected patients	Patients with intermediate/high risk (e.g., PAGE-B ≥ 10), advanced fibrosis, previous history of HCC, family history of HCC, or chronic HDV coinfection.
**MASLD without cirrhosis**	Low–intermediate	Not routinely recommended	HCC risk is variable; validated risk-stratification tools are lacking.
**Chronic HCV after SVR with advanced fibrosis**	Intermediate	Not routinely recommended	Residual risk persists, but evidence is currently insufficient to support universal surveillance.
**Other non-cirrhotic chronic liver diseases**	Variable	Individualized assessment	Routine surveillance is not currently recommended; further validation of risk prediction models is needed.

HCC, hepatocellular carcinoma; AFP, alpha-fetoprotein; HBV, hepatitis B virus; MASLD, metabolic dysfunction-associated steatotic liver disease; SVR, sustained virological response; HDV, hepatitis D virus; HCV, hepatitis C virus.

**Table 2:** The liver imaging reporting and data system (LI-RADS) criteria for imaging-based HCC diagnosis.

Imaging Modality	Major Imaging Features	Criteria for Definite HCC (LR-5) Diagnosis
**CT/MRI**	APHE (further categorized into rim and non-rim) Washout (peripheral and non-peripheral) Enhancing capsule Size Threshold growth (≥50% diameter increase in ≤6 months)	**Nodules ≥ 2 cm** Non-rim APHE and at least one additional major feature (non-peripheral washout, enhancing capsule, or threshold growth) **Nodules 10–19 mm** Non-rim APHE and either non-peripheral washout or threshold growth
**CEUS**	Size Rim APHE (non-rim and not peripheral discontinuous) Washout (refined into early <60 s or late ≥60 s, and into marked or mild)	**Nodules ≥ 10 mm** Non-rim and non-peripheral discontinuous APHE plus late and mild washout

HCC, hepatocellular carcinoma; APHE, arterial phase hyperenhancement; CT, computed tomography; MRI, magnetic resonance imaging; CEUS, contrast-enhanced ultrasound.

**Table 3:** HCC subtypes based on WHO 5th edition *.

HCC Subtype	Freq.	Key Clinical Features	Key Histopathology Features	Key Radiology Findings	Molecular Signature	Prognosis
**Fibrolamellar HCC**	1%	Young patients, no history of liver disease, normal AFP	Large eosinophilic neoplastic cells with prominent nucleoli; intratumoral fibrosis	Well-defined hepatic mass with a central fibrous scar and mixed enhancement in imaging	*DNAJB1-PRKACA* fusion	Comparable to HCC arising in a non-cirrhotic liver
**Macrotrabecular-massive HCC**	8.5–12% *	Linked to chronic HBV infection, elevated AFP levels	Trabeculae ≥ 10 cells thick, comprising over half the tumor	Non-uniform hypovascularity on arterial phase, with rim-like or focal areas of increased enhancement	TP53/ATM alterations, FGF19 gain, angiogenic gene upregulation	Reduced overall and disease- free survival; independent predictor of recurrence
**Steatohepatitic HCC**	10–20%	Predominant subtype; associated with metabolic dysfunction, chronic alcohol intake, diabetes mellitus type 2	Steatosis, hepatocyte ballooning degeneration, Mallory-Denk hyaline, lattice-like (“chicken-wire”) pericellular fibrosis, intratumoral inflammatory infiltrate	Imaging features reflecting intratumoral fat: increased US echogenicity, fat-equivalent CT density, T1-weighted MRI signal increase	Driven by IL-6/JAK/STAT signaling rather than WNT/β-catenin–TP53–TERT axis of classic HCC; serum CRP reflects pathway activity	Comparable to, or slightly better than conventional HCC
**Scirrhous HCC**	~4%	Behaves aggressively (vascular invasion, infiltrative growth, diminished survival)	Combination of mature hepatocyte-like and progenitor/stem-like cells embedded in dense fibrous stroma exceeding 50% of tumor volume	Rim-like peripheral uptake with gradual filling-in centrally, absence of washout, restricted diffusion in a target pattern; may resemble CCC	*TSC1/TSC2* alterations; activated TGF-β signaling	Not firmly established; overall survival similar to other subtypes, but recurrence at 2 years is higher than in non-scirrhous tumors
**Chromophobe HCC**	~3%	Mostly found in women; no identified risk factor	Pale/clear cytoplasm with otherwise bland cell appearance, scattered atypical nuclei; pseudocysts frequently form	No characteristic imaging pattern	Relies on the ALT mechanism; lacks *TERT* promoter alterations	Outcomes do not differ meaningfully from conventional HCC
**Clear-cell HCC**	3–7%	Underlying mechanisms not clarified yet	Predominantly clear cytoplasm (>80% of tumor cells), glycogen accumulation; steatosis must be ruled out	Imaging appearance overlaps with conventional HCC; no pathognomonic feature	*IDH1* alterations described in a subset	Generally good outcomes, though one cohort reported higher rates of relapse/spread
**Lymphocyte-rich HCC**	<1%	No connection with EBV; genomically microsatellite stable	Immune cell infiltrate—mostly CD8+ T cells—outnumbering tumor cells in over half the sections	No characteristic imaging pattern	Molecular drivers not yet characterized	Better outcomes relative to conventional HCC
**Neutrophil-rich HCC**	<1%	Signs of systemic inflammation, like increased leucocytes, CRP and IL-6	Heavy neutrophil infiltration within the tumor; high-grade cytology, sarcomatoid change sometimes	No characteristic imaging pattern	Tumor cells secrete G-CSF	Worse outcomes than typical HCC, with reduced survival

* In the 6th edition of the WHO classification of tumors of the digestive system, there are no changes in HCC histological subtyping [46,47]. HCC: Hepatocellular Carcinoma; WHO: World Health Organization; Freq.: Frequency; DNAJB1: Dna J Heat Shock Protein Family (Hsp40) Member B1; PRKACA: Protein Kinase cAMP-Activated Catalytic Subunit Alpha; AFP: A-Fetoprotein; HBV: Hepatitis B virus; TP53: Tumor Protein 53; ATM: Ataxia Telangiectasia Mutated; FGF19: Fibroblast Growth Factor 19; US: Ultrasound; CT: Computed Tomography; MRI: Magnetic Resonance Imaging; IL-6: Interleukin-6; JAK: Janus Kinase; STAT: Signal Transducer and Activator of Transcription; WNT: Wingless-related integration site; TERT: Telomerase Reverse Transcriptase; CRP: C-reactive protein; TSC1/TSC2: Tuberous Sclerosis Complex 1/Tuberous Sclerosis Complex 2; TGF-β: Transforming Growth Factor Beta; ALT: Alternative lengthening of telomeres; IDH1: Isocitrate Dehydrogenase 1; EBV: Epstein-Barr virus; G-CSF: Granulocyte Colony-Stimulating Factor.

## Data Availability

No new data were created or analyzed in this study. Data sharing is not applicable to this article.
